# Virulence Regulation and Drug-Resistance Mechanism of Fungal Infection

**DOI:** 10.3390/microorganisms10020409

**Published:** 2022-02-10

**Authors:** Letizia Angiolella

**Affiliations:** Department of Public Health and Infectious Diseases, Sapienza University of Rome, Piazzale Aldo Moro, 5, 00100 Roma, Italy; letizia.angiolella@uniroma1.it

In this issue, I propose promising developments in the field of the mechanism of virulence factors that can be linked to antifungal resistance. Fungi are among the most widespread eukaryotic microbes on earth and can cause crop losses and the elimination of various life forms. In addition to being present in the background, many fungal species are part of the microbiota and are present in various anatomical sites including the skin, lungs, genitourinary, and oral and gastrointestinal tract, where they play an important part in human health. Fungal diseases are generally called mycoses. Some fungi, primarily pathogens, if inhaled in large quantities can cause diseases in immunocompetent hosts. Commensal fungal species can become invasive pathogens when the immune system is compromised, thus causing systemic mycosis which affects multiple organs and systems.

Very often undervalued as a cause of infection in humans, fungi are involved in about 1.5 million deaths and 1.7 billion superficial infections each year, thus representing a serious economic problem. In addition to causing acute or chronic infections, some studies have shown a correlation between fungal colonization and the progression of diseases such as pancreatic cancer and alcoholic cirrhosis [1].

Fungi can cause diseases by using their virulence factors to enable them to live in the hostile environment of the host. These factors include their ability to adhere to the host’s tissues, to release enzymes capable of harming the tissue, and to directly interfere with the host’s defenses. In addition, some fungi are dimorphic; they have the ability to switch from one shape to another. Their ability to live in temperatures up to 37 °C, referred to as thermotolerance, is vital for their survival in their hosts and makes their diffusion possible.

## 1. Major Virulence Factors

### 1.1. Adhesion of the Cells

Some virulence factors consist of structures that allow fungi to adhere to tissues and prevent them from being removed by ciliary actions or slimy membranes. *C. albicans* for instance, has several adhesion molecules, and can produce a biofilm increasing its pathogenicity. The main bond molecules are Als, Hwp1p, Eap1p, Cshlp, and others. The proteins mediating the adhesion to collagen, laminin, endothelial cells, epithelial cells and cell-to-cell aggregation are coded by eight genes. Hwp1p is able to bind to the epithelium while Int1p adheres to platelets. *A. fumigatus*, *H. capsulatum*, and *P. brasiliensis* are pathogenic fungi with adhesion molecules. A characteristic of *A. fumigatus* is having conids that are covered with hydrophobic proteins, which are coded for by the RODA and RODB genes and facilitate adhesion to albumin and collagen. On the superficial hyphae, there are receptors such as galactomannan and chitin that mediate complement adhesion, fibrinogen, immunoglobulin, and surfactants A and D. Blastomyces modulates the host immune response binding to CR3 and CD14 receptors present on phagocyte. Other fungi such as *H. capsulatum*, *P. brasiliensis* and Coccidioides use other receptors for adhesion to the host.

### 1.2. Dimorphism

As mentioned above, many pathogenic fungi have dimorphism (that is, they can shift from a non-pathogenic form or as commensals to a pathogenic form capable of causing mycoses). *B. dermatitidis*, *C. immitis*, *H. capsulatum*, *P. brasiliensis,* and *C. albicans* are dimorphic fungi. They may be yeasts or molds; yeasts have either round or ovoid cell, which divide by binary fission producing a distinct and independent daughter cell. On the other hand, molds are filamentous grown by apical extension, creating cell separated by septa which can be branched but united to the mold (which takes the name of hyphae or mycelium). Some fungi may have additional morphotypes. For example, *C. immitis* can form large endosporular spheres. Finally, there are transitional forms such as pseudohyphae as in *C. albicans*.

### 1.3. Thermotolerance

Another important virulence factor for fungi causing systemic infections is their ability to grow at elevated temperatures over 37 °C. For example, *A. fumigatus* can grow up to 55–77 °C. The ability to adapt to growing at high temperatures in fungi depends on an HSP of 70 [2].

Temperature-pathogenic fungi switch from one form to another at different temperatures, and in fact most fungi look like molds at room temperature. They turn into yeasts at the temperature of the host, which is the pathogenic form. In *H. capsulatum*, when the transition from mycelia to yeast is blocked, the organism continues to grow at 37 °C. However, it is not virulent. For *C. albicans*, however, both forms are pathogenic and the transition from one form to another depends on environmental changes in both temperature and pH. The hyphal shape makes tissue invasion possible.

### 1.4. Presence of Capsule

Some pathogenic fungi may have a capsule. *C. neoformans* has a glucoronoxymannan capsule enabling it to resist phagocytosis. Generally, polysaccharide capsules are very evident in strains (causing infections), whereas in the environment the capsule is less present.

In fact, strains without capsules are avirulent as they can be engulfed. The genes expressing the capsule are CAP 59 and CAP 64. Capsules are able to reduce complement and cause deregulation of cytokine release. Capsule also prevent the mobilization of leukocytes in the infected area. Pathogenic fungi are able to release degrading enzymes that allow them to spread and cause disease, damaging tissues and compromising the host’s immune system.

### 1.5. Release of Enzymes

*C. albicans* secretes extracellular phospholipases, lipases, and proteases in greater amounts when it is pathogenic. Phospholipases A, B, C, and D break the external links and are vital for nutrition and iron retrieval. *C. albicans* also secretes SAP (aspartil proteinase secreted) which hydrolyzes extracellular matrix proteins, clotting factors, host defense proteins such as mucin, IgA, and lactoferrin, and other complements. *A. fumigatus* also secretes proteases and phospholipases that destroy elastin in the lung tissue. Serine proteases damage collagen, fibrin, and fibrinogen. *C. neoformans* also secretes proteases and phospholipases, lysophospholipases, and lysophospholipase-transacilase (LPTA). All of these proteases can destroy the pulmonary surfactant and increase adhesion. In addition, some studies indicate that *C. neoformans* invades the CNS with the production of ureases. Even Coccidioide uses urease to increase alkalinity in certain infected areas.

Pathogenic fungi can produce enzymes which neutralize the toxic oxygen (ROS) released by neutrophils and macrophages. *C. albicans* uses superoxide dismutase and HSP for protection against ROS, while *C. neoformans* produces copper, zinc, and peroxidase to resist oxidation. *A. fumigatus* produces some catalases, namely Cat-A (associated with conids) and Cat 1p and Cat 2p (associated with hyphae and superoxide dismutase), which defends it from oxidative damage. In addition, some fungi produce melanin that protects them against severe conditions such as UV radiation, temperature rises, and ROS. Also, in *C. neoformans*, melanin permits one to avoid the damage of antifungal substances and inhibit antibody-mediated phagocytosis. Melanin is also synthesized by *A. fumigatus*, *H. capsulatum*, Blastomyces, and *P. brasiliensis.*

### 1.6. Iron Acquisition

Fungi need iron to breathe, grow, and for many other metabolic processes. However, iron does not exist in the free form in the host since it is usually bound to proteins. *A. fumigatus* utilizes three Fe uptake mechanisms: Fe reductase uptake, siderophoro-mediated Fe uptake, and ferrous Fe uptake mechanisms. In *A. nidulans*, two major siderophores triacetylfusannin C (TAFC) and desferriferricrocin (DFFC) have been identified. *C.albicans* obtains iron by siderophores, or through absorption from heme into erythrocytes using hemoglobin receptors on the cell surface. *C. albicans* also uses a reduction mechanism.

### 1.7. Other Systems

*A. fumigatus* releases a number of toxins such as aflatoxin and gliotoxin. The aflatoxin produced by *A. fumigatus* is not a virulence factor; it is toxic for hepatocyte and induces the carcinogenesis. Gliotoxin inhibits the immune system and phagocytosis through macrophages and activation of T cells. It also reduces ciliary movement and causes injury to the epithelium. Most other fungi release secondary metabolites that have numerous cellular activities, some of which are probably significant in pathogenesis.

Calcineurin is a control device for pathogenic fungi. It may affect the manifestation of several virulence factors. The calcineurin is vital for the growth of *A. fumigatus* and contributes to tissue attack. In CNS infections *C. neoformans* uses mannitol to defend fungi from oxidative damage. It is produced in great quantities and can contribute to cerebral edema [3].

## 2. Antifungal Drugs

While there are many classes of antibiotics available for bacterial infections, antifungal drugs are limited in number and mechanism of action and belong to three main classes, including azoles (fluconazole, itraconazole, voriconazole, posaconazole, etc.), echinocandins (caspofungin, micafungin and anidulafungin), and polyenes (such as amphotericin B (AMB)).

Azoles block the production of ergosterol, a key constituent of the fungal cytoplasmic membrane, bind to Erg11 in *Candida* and Cyp51A in *Aspergillus* species, while echinocandins affect the catalytic subunit of the enzyme β-1,3-D-glucan synthase (coded by the FKS genes) and inhibit the production of β-1,3-D-glucan (a structural constituent of the cell wall). Finally, polyenes bind directly to the ergosterol of the cell membrane and causes the formation of great pores on the cell membrane, resulting in cell death. Before an overuse of antifungal drugs, epidemiological studies showed that most fungal species were sensitive to all classes of antifungal drugs.

## 3. Resistance to Drugs

The extensive use of these drugs has changed the epidemiological scenario of infections, where many fungi show resistance to one and/or more classes of antifungal agents, often causing a therapeutic failure. One important clinical problem is drug resistance. This is principally due to an increase in patients at risk of invasive mycosis infections due to intricate surgical procedures, immunosuppression, or reduced immune function. The current emergence of fungi resistant to other than one class of antifungal drugs is of serious concern, particularly as it mostly affects fungal species infecting humans.

### 3.1. Resistance to Azole

Efflux pumps are involved in resistance to azole drugs and belong to two categories, the ATP-binding cassette (ABC) transporters (CDR1 and CDR2) and the major trans superfamily facilitators (MFS).

It represents the most widespread mechanism of azole resistance, causing an increase in the action of the drug efflux pump due to increased functional alterations in transcription factors regulating ATP (ABC) cassette transporters (TAC1 in *C. albicans* and PDR1 in *C. glabrata*) and the main facilitated superfamily pumps (MRR1 in *C. albicans*). When fungi are exposed to azoles, overexpression of efflux pumps is a principal response. It should be noted that several of these transcription factors similarly control other genes that contribute to the resistance, virulence, or suitability of yeast cells [4].

Azoles have fungistatic activity against Candida, and they react by binding and inhibiting the intracellular objective enzyme ERG11p involved in ergosterol biosynthesis. Besides, more than 140 modifications in the ERG11 target gene have been reported, some of which originate completely in azole resistant isolates, while others were also present in susceptible isolates. The influence of the alteration on azole resistance depends on the site and precise exchange of an amino acid. Many mutations of the target occur simultaneously and may also affect other resistance mechanisms.

The ATP (ABC) class includes the main azole drug transporters CDR1, CDR2, and *C. glabrata* (as well as CgSNQ2), and confers resistance to panazole. The MDR1 transporter belonging to the major facilitator superfamily (MFS) is also implicated in the azole resistance of Candida but not in the resistance to posaconazole, itraconazole, or isavuconazole. Specific regulators TAC1 for CDR1 and CDR2 and MRR1 for the regulation of MDR1 expression determine the expression levels of drug transporters. Since the different efflux pumps have similar functions, alterations such as the damage to a single gene of a function encoding an efflux pump or the increased function of less efficient efflux pumps result in uncertain effects on resistance, while more important alterations are due to overexpression combined with homozygosity. Alterations in the targets of the antifungal drug, due to increased expression of the target or changes within the target protein sequence, similarly mediates the resistance of the antifungal drug. Erg11, which is the target of azoles, is a lanosterol 14α-demethylase dependent on cytochrome P450 needed for ergosterol biosynthesis. Increased expression of ERG11 is also observed in naturally resistant non-*C. albicans* species. However, the mechanism underlying this is still unidentified. Changes in ERG11 have also been detected in *Candida* spp. and over 100 different ERG11 alleles have been labeled. [5].

### 3.2. Resistance to Echinocandin

Echinocandin drugs (caspofungin, micafungin, and anidulafungin), permitted for clinical use in 2001, affect and constrain β-1-3-D-glucan synthase and block the biosynthesis of β-1,3-glucan, an essential constituent of the fungal cell wall. They are active and fungicidal against *Candida* species. The enzyme complex contains a structural/catalytic subunit coded by FKS genes; its activity is controlled by Rho, a GTP-binding protein. Clinical resistance includes alteration of Fks subunits. In *C. glabrata*, two genes, FKS1 and FKS2, code the catalytic subunits of glucanosintase. In the greatest *Candida* species, alterations happen in two highly preserved regions of FKS1 and, in *C. glabrata*, FKS2 amino acid switches produce high MIC values and major changes can decrease susceptibility to the drug by over 3000-fold. [6] A single mutation is enough to induce drug resistance, although in some cases it is possible to find different alterations. The level of resistance depends on the precise codon involved, the specific change, and the species in which it occurs.

### 3.3. Resistance to Polyenes

Amphotericin B also has a fungicidal activity by binding to ergosterol in the fungal cell membrane. After binding, a pore forms in the membrane, causing the loss of intracellular compounds and cell death. The precursor of ergosterol is lanosterol. Biosynthesis consists of a series of enzymatic phases all determined by the ERG genes (ERG6, ERG11, ERG24, ERG25, ERG26, ERG27, ERG2, ERG3, ERG5, and ERG4). Resistance to amphotericin B is an occasional phenomenon in *Candida* spp. (1–3%).

### 3.4. Resistance to Flucytosine

Flucytosin is actively transported into the fungal cell by permease (encoded by FCY2). It is transformed into 5-fluorouracil or5-fluorouridine monophosphate by the enzymes cytosine deaminase or uracil phosphoribosyl transferase coded by the FCY1 and FUR1 genes, respectively, and acts by inhibiting transcription, DNA replication, and protein synthesis. Resistance emerges fast when used alone and has been attributed to alterations in the FCY2, FCY1, and FUR1 genes. In addition, three novel biological procedures affecting flucitosin resistance in *C. glabrata* have been identified: arginine homeostasis, cell wall remodeling, and Fps aquaglyceroporins.

In this analysis, the contributions from leading authors provide the following:

1. A comprehensive update on virulence factors to protect against fungal infections;
2. Molecular studies highlighting the relations between virulence factors and drug resistance;
3. Studies describing the inhibition of virulence factors which can be used to overcome drug resistance;
4. Research of innovative targeted compounds to contrast antifungal infections.

This collection of reports will provide information of these virulence factors important for the research of new drugs active in the prevention and treatment of fungal diseases.
